# Circulating Biomarkers in Advanced Colorectal Cancer Patients Randomly Assigned to Three Bevacizumab-Based Regimens

**DOI:** 10.3390/cancers6031753

**Published:** 2014-08-29

**Authors:** Antonia Martinetti, Rosalba Miceli, Elisa Sottotetti, Maria Di Bartolomeo, Filippo de Braud, Arpine Gevorgyan, Katia Fiorella Dotti, Emilio Bajetta, Manuela Campiglio, Francesca Bianchi, Giacomo Bregni, Filippo Pietrantonio

**Affiliations:** 1 Medical Oncology Department, Fondazione IRCCS Istituto Nazionale dei Tumori, Via G. Venezian, Milan 1-20133, Italy; E-Mails: elisa.sottotetti@istitutotumori.mi.it (E.S.); maria.dibartolomeo@istitutotumori.mi.it (M.D.B.); filippo.debraud@istitutotumori.mi.it (F.B.); arpine.gevorgyan@istitutotumori.mi.it (A.G.); katia.dotti@istitutotumori.mi.it (K.F.D.); giacomo.bregni@gmail.com (G.B.); filippo.pietrantonio@istitutotumori.mi.it (F.P.); 2 Medical Statistics, Biometry and Bioinformatics Unit, Fondazione IRCCS Istituto Nazionale dei Tumori, Via G. Venezian, Milan 1-20133, Italy; E-Mail: rosalba.miceli@istitutotumori.mi.it; 3 Medical Oncology Unit, IDO Policlinico di Monza, Via Amati, Monza 111-20900, Italy; E-Mail: emilio.bajetta@policlinicodimonza.it; 4 Department of Experimental Oncology, Fondazione IRCCS Istituto Nazionale dei Tumori, Via G. Venezian, Milan 1-20133, Italy; E-Mails: manuela.campiglio@istitutotumori.mi.it (M.C.); francesca.bianchi@istitutotumori.mi.it (F.B.)

**Keywords:** bevacizumab, colorectal cancer, biomarkers, angiogenesis, VEGF, CEA, SDF-1

## Abstract

The need to identify biomarkers for bevacizumab-based treatment in advanced colorectal cancer is imperative. The aim of this study was to investigate the prognostic role of circulating VEGF, PDGF, SDF-1, osteopontin and CEA in patients randomly assigned to three bevacizumab-based regimens. Plasma samples from 50 patients treated at a single Institution were analysed using the multiplex assay BioPlex™ 2200 (Bio-Rad Laboratories, Inc, Berkeley, CA, USA) at baseline, before first three cycles and subsequently every three cycles until disease progression. Prognostic analyses of baseline values were performed using multivariable Cox models, including disease extension >10 cm or ≤10 cm (measured as the sum of the diameters for all target lesions) as adjustment factor. The association between progression-free and overall survival and biomarkers modulation during treatment was studied using multivariable Cox models, which included summary statistics synthesizing during-treatment modulation together with disease extension. The biomarkers significantly associated with disease extension were baseline CEA (*p =* 0.012) and SDF-1 (*p =* 0.030). High values of VEGF and SDF-1 tended to be associated with worse prognosis, especially in terms of overall survival. The negative prognostic trend was more marked for baseline CEA as compared to other biomarkers; increasing values during treatment was significantly related to worse prognosis independently of disease extension (*p =* 0.007 and 0.016 for progression-free and overall survival, respectively). VEGF is related to bevacizumab pharmacodynamics and is associated to other angiogenic cytokines; some of the proposed biomarkers such as SDF-1 and CEA should be further validated for prognosis assessment and monitoring of bevacizumab-based treatment of advanced colorectal cancer.

## 1. Introduction

Angiogenic process play a key role in colorectal cancer growth, invasiveness and metastatic spread *in vitro* and *in vivo* [1]. In metastatic colorectal cancer (mCRC) angiogenesis can be sustained by redundant pathways, with both autocrine and paracrine mechanisms, ultimately leading to spouting of new vessels from pre-existing ones. Since angiogenesis is regulated by the balance between promoting and inhibitory factors, it must be pointed out that metastatic potential is gained through upregulation of several stimulating molecules, primarily vascular endothelial growth factor (VEGF), but also basic fibroblast growth factor (bFGF), platelet-derived growth factor (PDGF) and placenta growth factor (PIGF) [2]. The therapeutic blockade of VEGF induce complex changes in the stromal compartment of tumor lesions, such as reorganization of chaotic blood vessels, vasculature pruning and reduction of interstitial fluid pressure, allowing a better intratumoral delivery of chemotherapeutic drugs [3,4].

Bevacizumab (BEV) is a fully humanized monoclonal antibody which binds soluble VEGF and prevents the stimulation of its receptors on endothelial cells [5]. Antiangiogenic treatment with BEV combined with standard chemotherapy was shown to prolong survival of mCRC patients, leading to approval for first or second line treatment in association with fluoropyrimidine-based chemotherapy [6,7,8]. BEV demonstrated also to confer an increase of response rate, with interesting applications in patients with inoperable liver disease and potential conversion to resectability with increase of pathological complete responses [9,10].

Primary refractoriness or acquired resistance to BEV usually develop and constitute a relevant issue in clinical practice. Several mechanisms of resistance to antiangiogenic treatment have been explored, including tumor infiltration by myeloid cells producing proangiogenic ligands, activation of VEGF-independent angiogenic pathways due to overexpression of alternate mediators, *in-situ* tissue expression of angiogenesis-related markers (such as VEGF, PDGF-C, delta-like 4 (DLL4) and Neuropilin-1) and polymorphisms in the VEGF signalling [11]. Given the significant medical burden of CRC and the socio-sanitary costs of monoclonal antibodies, the identification of predictive biomarkers of antiangiogenic treatment efficacy is a priority for oncologists. In this regard, the measurement of circulating biomarkers is particularly attractive due to low costs, good reproducibility, accessibility and rapid quantification of results; nevertheless, the validation of predictive biomarkers of both efficacy and resistance remains elusive.

In this retrospective analysis we assessed the role of several circulating biomarkers in a subgroup of patients with mCRC who were enrolled into an Italian, multicenter, phase II trial evaluating three different BEV-containing first-line regimens [12]. In particular, we explored the association of the baseline circulating levels of VEGF, PDGF-AA, PDGF-AB/BB, stromal cell-derived factor-1 (SDF-1), osteopontin (OPN) and carcinoembryonic antigen (CEA) with tumor burden as shown by radiologic assessment, and with clinical benefit from BEV as measured by progression-free survival (PFS) and overall survival (OS). Secondly, we investigated the association between biomarkers modulation during treatment and the two end-points (PFS and OS).

## 2. Experimental

### 2.1. Patients

We analysed the blood samples from 50 mCRC patients treated within a multicenter, randomized, 3-arms, phase II trial as follows: arm A, capecitabine 2000 mg/mq daily from day 2 to 15 and irinotecan 240 mg/mq plus BEV 7.5 mg/kg on day 1 every 3 weeks; arm B, capecitabine 2500 mg/mq from day 1 to 14 plus BEV 7.5 mg/Kg on day 1 every 3 weeks; arm C, metronomic capecitabine at continuous daily dosing of 1300 mg/mq plus BEV 7.5 mg/Kg on day 1 every 3 weeks. Patients in arms A and B received up to 6 cycles and, in case of objective response or disease stabilization, were treated with maintenance BEV alone until progressive disease (PD); patients in arm C received treatment until PD. Treatment was also discontinued in case of unacceptable toxicity or informed consent withdrawal. All patients analysed in the present ancillary protocol were enrolled at our Institution, the National Cancer Institute of Milan. Eligible patients were ≥18 years old, with an Eastern Cooperative Oncology Group (ECOG) performance status of 0 or 1 and were affected by histologically confirmed mCRC, with at least one unidimensionally measurable lesion, no prior chemotherapy or a time interval >6 months from adjuvant treatment completion. All patients had adeguate hepatic, renal and bone marrow functions. Main exclusion criteria included the presence of central nervous system metastases, history of inflammatory bowel disease or acute/subacute bowel occlusion, serious non-healing wounds or ulcers, evidence of bleeding diathesis or coagulopathy, uncontrolled hypertension, clinically significant or active cardiovascular disease, treatment with full-dose anticoagulants or aspirin (>325 mg daily) or other medications known to predispose to gastrointestinal ulceration. 

Written informed consent was obtained from each patient for both the main study and the ancillary biomarkers protocol.

To evaluate disease extension for each patient the sum of the longest diameters for all target lesions was calculated at baseline according to RECIST criteria. Patients were categorized in two groups according to disease extension if the above-mentioned sum was >10 cm or ≤10 cm [13].

### 2.2. Statistical Methods

Standard descriptive statistics were calculated for categorical data (*i*.*e*., absolute frequency and percentage) and continuous data (*i*.*e*., median and interquartile (IQ) range), as listed in the tables. The degree of association between pairs of biomarkers measured at baseline was assessed using the Spearman correlation coefficient. Kruskal-Wallis or Mann-Whitney tests were performed to assess baseline biomarker differences between groups. 

Further analyses were conducted studying a biomarker at a time; no joint analyses were performed. CEA was analyzed in a log scale, due to the high positive asymmetry of its distribution.

The prognostic end-points studied were PFS and OS. PFS time was computed as the interval between the date of randomization and the date of disease progression or death due to any cause, whichever occurred first, or censored at the date of last follow-up assessment for alive and progression-free patients. OS time was computed as the interval between the date of randomization and the date of death due to any cause, or censored at the date of last follow-up assessment for alive patients. PFS and OS curves were estimated by Kaplan Meier method and statistically compared by using the log-rank test. 

Multivariable Cox regression models were fitted for investigating the association between PFS or OS and biomarker baseline values independently of disease extension, which was included in the model as adjustment factor because of its correlation with the biomarkers. With the aim of exploring in detail the relationship between the risk of progression or death and the biomarkers, we performed Cox analyses in which the biomarkers were modeled as continuous variables by using three-knot restricted cubic splines [14]. Moreover, being well aware that any variable categorization implies loss of information, additional Cox analyses were performed modeling the biomarkers as three categories variables using the tertiles of their distribution as pre-determined cut off values. This represents a compromise between not using too much degrees of freedom in the model (given the low number of events) and giving an idea of the complex prognostic associations highlighted by means of restricted cubic spline modeling. 

Further analyses were aimed at studying the biomarker modulation during treatment (longitudinal pattern). Descriptive analysis was performed by plotting individual biomarkers levels *versus* detection time. Multivariable mixed effects models were also fitted for testing the influence of treatment arm, disease extension and, alternatively, the PFS or OS status on biomarker values during treatment. Time was modeled by means of a three-knot restricted cubic spline [15], and all the corresponding coefficients were included both as fixed and random effects. 

We also studied the association between PFS or OS and the biomarker longitudinal pattern using multivariable Cox models including summary statistics, suitable for synthesizing the longitudinal pattern, and disease extension as adjustment factor. The strategy was the following: for each biomarker we fit a set of mixed effects models including detection time as the only covariate; the models differed according to the way of modeling time, *i*.*e*., by means of: 1st, 2nd and 3rd degree polynomials, a three-knot restricted cubic spline [14], and one-knot linear splines with knots varying within the time range. The “best” model was chosen based on the Akaike Information Criterion [15] (the lower the better). For all the biomarkers the one-knot linear spline model was chosen as best one; the knot was positioned at end of cycle 1 for all the biomarkers but CEA, for which the knot was chosen at end of cycle 3. We took into consideration the following summary statistics derived from the one-knot linear spline model: the first slope, synthesizing the biomarker pattern in the interval baseline-first cycle, and the second slope, synthesizing the biomarker pattern in the subsequent interval. 

We considered as significant two-sided *P* values below the 5% conventional threshold. All statistical analyses were performed using the SAS (SAS Institute Inc., Cary, NC, USA, 2000) and the R software (R Development Core Team, R Foundation for Statistical Computing, ISBN 3-900051-07-0, Vienna, Austria, 2006). R: A language and environment for statistical computing [16].

### 2.3. Plasma Sample Collection and Analysis

Blood samples were withdrawn at baseline, before the first three cycles and subsequently every three cycles concomitantly with radiologic reassessment, as well at PD (when available). In order to obtain the plasma sample for angiogenic factors assays blood was collected in BD vacutainer^®^ EDTA tube that was immediately put in an ice bath and centrifuged at 1500× *g* at 4 °C within 1 h from collection to avoid cytokines degradation. The supernatant obtained was aliquoted and stored at −80 °C until analyses. Bio-Plex™ 2200 system human assays (Bio-Rad Laboratories, Hercules, CA, USA and Millipore, Boston, MA, USA) for simultaneous quantification of angiogenic factors VEGF, PDGF-AA, PDGF-AB/BB, SDF-1 and OPN were used according to the manufacturer instructions. Briefly, plasma samples were incubated on 96-well plate with polystyrene-beads coated with angiogenic factor-specific antibodies, and then exposed to detection antibodies prior incubation with Strepatavidin-PE. Capture lysates were analysed on the Bio-Plex™ 2200 system. All samples were assayed in duplicate. The intra-assay % coefficient of variations (CVs) were: VEGF 7%, OPN 8%, SDF-1 6%, PDGF-AA and PDGF-AB-BB 2%–13% respectively. The inter-assay % CVs were: VEGF 9%, OPN 8%, SDF-1 6%, PDGF-AA and PDGF-AB-BB 5%–19% respectively. CEA serum level was measured at baseline and every three cycles concomitantly with radiologic reassessment, as well at PD (for all patients). To obtained the serum sample for CEA assay blood was collected in BD vacutainer^®^ separator tube and was allowed to clot for 30 min at room temperature (RT) and then centrifuged at 1500× *g* at RT. The assay was conducted by immunoradiometric assay (IRMA) CEA IRMA CT supplied by Radim (Rome, Italy) according to manufacturer instructions. CEA normal value was <5 ng/mL.

## 3. Results

### 3.1. Patients

Baseline patients characteristics are shown in Table 1. At 36 months, PFS was 7.7% (95% confidence interval (CI), 1.2–50.6) in arm A, 0% in arm B and 13.3% (95% CI, 3.7–48.4) in arm C (*p =* 0.664). The corresponding estimates for OS were 23.1% (CI, 8.6‑62.3) in arm A, 31.8% (CI, 17.3%–58.7%) in arm B and 16.7% (CI, 5.0%–56.1%) in arm C (*p =* 0.845).

**Figure 1:**
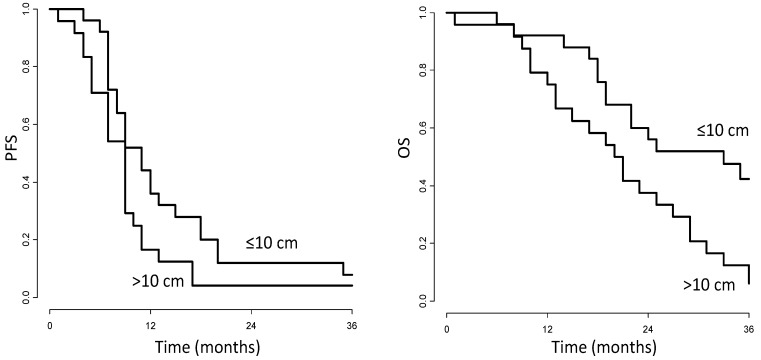
Kaplan-Meier progression-free survival (PFS, left panel) and overall survival curves (OS, right panel) according to disease extension (sum of the longest diameters of target lesions ≤10 or >10 cm, respectively) in patients treated with bevacizumab containing regimens.

Figure 1 shows the PFS and OS curves according to disease extension (≤10 cm and >10 cm). At 36 months, PFS was 8.0% (CI, 2.1%–30.2%) for patients with disease extension ≤10 cm and 4.2% (CI, 0.6%–28.4%) for patients with disease extension >10 cm (*p =* 0.056); OS was significantly different in the two groups: 42.4% (CI, 26.4%–68.0% ) and 6.3% (CI, 1.1%–35.7%) (*p =* 0.011).

**Table 1:** Patients characteristics at baseline.

Baseline Characteristics	Number of Patients (%)
Gender:	
Male	29 (58)
Female	21 (42)
Age:	
Median (interquartile range)	60 (51–68)
Arm:	
A	13 (26)
B	22 (44)
C	15 (30)
Perfomance Status:	
0	48 (96)
1	2 (4)
Disease Extension: *	
>10 cm	25 (51)
≤10 cm	24 (49)
Primary lesion:	
Colon	33 (66)
Rectum	17 (34)
Number of metastatic sites:	
1	37 (74)
>1	13 (26)

* One patient has no evaluable lesions.

### 3.2. Biomarkers Baseline Level

Table 2 shows the results of the analysis of pair association between biomarkers. In general, the association levels were weak; the highest and significant positive association was highlighted between VEGF and SDF-1 (Spearman correlation coefficient, ρ = 0.74) and between PDGF-AB/BB and PDGF-AA isoforms (ρ = 0.75). The latter biomarker was significantly negatively associated with OPN (ρ = −0.29) and positively with CEA (ρ = 0.28); however, the degree of association was fairly low in both cases. There was no significant difference in basal biomarker levels between treatment arms (data not shown).

Table 3 shows descriptive statistics of baseline biomarker distribution according to disease extension. In general, greater disease extension was associated with higher biomarker levels. As regards VEGF, PDGF isoforms and OPN, no significant difference could be found according to disease extension, whereas for SDF-1 and CEA the between groups difference reached statistical significance (*p =* 0.030 and 0.012, respectively).

**Table 2:** Association between biomarker baseline values.

Biomarker	OPN	PDGF-AB/BB	PDGF-AA	SDF-1	CEA
VEGF (pg/mL)	0.18 (0.210)	0.08 (0.569)	−0.02 (0.888)	0.74 (<0.001)	0.21 (0.197)
OPN (pg/mL)	-	−0.02 (0.871)	−0.29 (0.045)	0.16 (0.273)	−0.01 (0.949)
PDGF-AB-BB (pg/mL)	0.08 (0.569)	-	0.75 (<0.001)	0.15 (0.319)	0.25 (0.127)
PDGF-AA (pg/mL)	−0.29 (0.045)	0.75 (<0.001)	-	−0.01 (0.943)	0.28 (0.084)
SDF-1 (pg/mL)	0.16 (0.273)	0.15 (0.319)	−0.01 (0.943)	-	0.13 (0.442)
CEA (ng/mL)	−0.01 (0.949)	0.25 (0.127)	0.28 (0.084)	0.13 (0.442)	-

The Table shows the estimates of the Spearman correlation coefficient and, in parenthesis, the corresponding *p* value for testing null hypothesis of no association.

**Table 3:** Biomarker distributions at baseline according to disease extension.

Biomarker	≤10 cm		>10 cm		P
VEGF (pg/mL)	132.90	(38.89–218.70)	203.20	(93.28–277.50)	0.183
OPN (pg/mL)	6.17	(2.86–10.87)	7.51	(6.41–8.31)	0.217
PDGF-AB/BB (pg/mL)	27470	(15,480–41,810)	33,460	(18,450–46,600)	0.480
PDGF-AA (pg/mL)	23620	(17,860–33,420)	26,880	(18,660–51,000)	0.258
SDF-1 (pg/mL)	75.23	(34.35–94.53)	98.85	(74.22–140.00)	0.030
CEA (ng/mL)	5.60	(2.77–13.88)	32.76	(5.76–2080.00)	0.012

The Table shows the median values and, in parenthesis, the interquartile range; P: *p* value at Mann-Whitney test.

**Figure 2:**
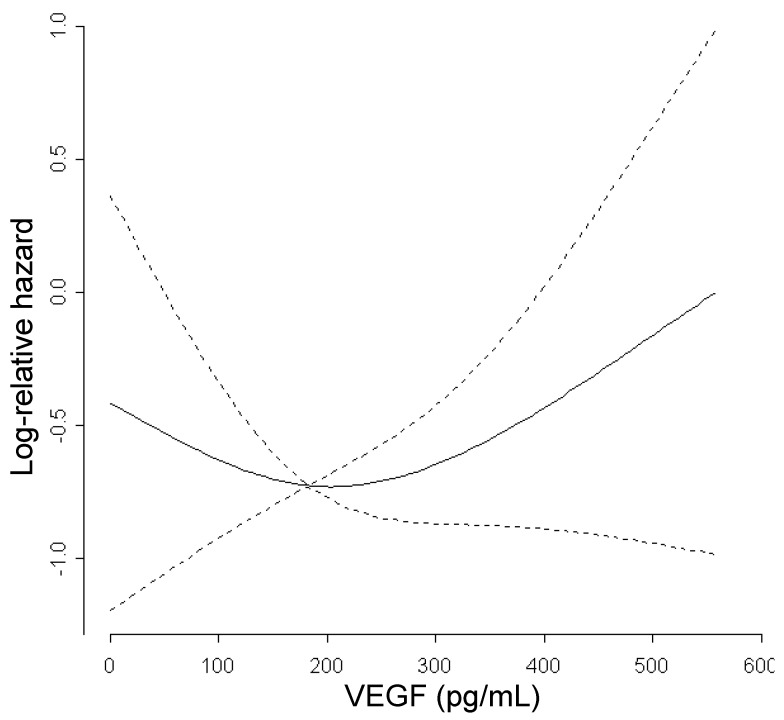
Relationship between the risk of death and baseline VEGF levels. The risk of death was measured by the log-relative hazard from the multivariable Cox model for OS. Continuous line: log-relative hazard plot; dotted lines: 95% log-relative hazard confidence bands.

In the multivariable Cox analyses of outcome, when analyzing the biomarkers as continuous variables by means of restricted cubic splines, including disease extension as adjustment factor, almost all them exhibited a complex relationship with the risk of progression or death; however, no significant results were obtained for both PFS and OS (data not shown). Taking VEGF as an example (Figure 2), we found that the risk of death decreased at increasing VEGF up to about 200 pg/mL (approximately the median value) and increased thereafter; comparing VEGF values as high as 600 pg/mL (approximately the maximum value) *versus* 200 pg/mL, the risk of death was about two-fold (HR = 2.33; 95% confidence interval: 0.75–7, 22). Being the spline modeling not familiar to the great majority of readers, we tried a way for “translating” the continuous prognostic trends in more comprehensible language by using a three-tires biomarker categorization system, as shown in Table 4. The table reports the results of the multivariable Cox models of PFS and OS, again showing that none of the biomarkers was significantly associated with prognosis; only SDF-1 showed a trend towards significance for OS, but did not reach the pre-specified significance limit of 5%. High values of angiogenesis-related biomarkers (VEGF, OPN, PDGF isoforms and SDF-1) were associated with a worse prognosis, especially in terms of OS. The highest HR values were obtained for VEGF, for which worse prognosis was associated with high levels (HR for comparing upper-tertile class *versus* intermediate class = 1.73 for PFS and 1.98 for OS). However, intermediate class patients had a lower risk of progression or death than lower class patients, as indicated by HRs values less than one. The above HR estimates are well translating the Figure 2 relationship. As shown in Table 2, VEGF was highly positively correlated with SDF-1; thus, as expected, the latter exhibited similar prognostic trends as those observed for VEGF. Baseline CEA, as well, was not significant but, as compared with the other biomarkers, the negative prognostic trend at higher values was more marked, as denoted by the HR estimates greater than 1 for both the comparisons of Intermediate *versus* Lower class and Upper *versus* Intermediate class. Such a result means that the prognosis is worsening at increasing CEA levels, whereas for the other angiogenesis-related biomarkers, such as VEGF, only very high values were associated with significant risk (Table 4).

**Table 4:** Results from multivariable Cox models for studying the association between progression-free survival or overall survival and baseline biomarker levels.

Biomarker	Progression-Free Survival			Overall Survival		
	HR	CI	P	HR	CI	P
VEGF						0.147
Intermediate class *versus* Lower class	0.49	(0.23,1.02)	0.141	0.45	(0.20,1.05)	
Upper class *versus* Intermediate class	1.73	(0.82,3.62)		1.98	(0.85,4.57)	
OPN						0.349
Intermediate class *versus* Lower class	0.89	(0.42,1.89)	0.948	0.66	(0.27,1.63)	
Upper class *versus* Intermediate class	1.01	(0.49,2.07)		1.76	(0.81,3.82)	
PDGF-AB/BB						0.602
Intermediate class *versus* Lower class	1.04	(0.51,2.16)	0.709	0.81	(0.37,1.80)	
Upper class *versus* Intermediate class	0.74	(0.35,1.56)		0.80	(0.35,1.83)	
PDGF-AA						0.408
Intermediate class *versus* Lower class	0.75	(0.36,1.58)	0.397	0.69	(0.30,1.61)	
Upper class *versus* Intermediate class	1.80	(0.77,4.22)		1.81	(0.75,4.33)	
SDF-1						0.060
Intermediate class *versus* Lower class	0.56	(0.27,1.17)	0.276	0.39	(0.18,0.88)	
Upper class *versus* Intermediate class	1.17	(0.56,2.45)		1.31	(0.57,2.99)	
CEA						0.323
Intermediate class *versus* Lower class	1.36	(0.59,3.10)	0.552	1.81	(0.70,4.64)	
Upper class *versus* Intermediate class	1.15	(0.50,2.64)		1.05	(0.44,2.50)	

The biomarkers were modelled as categorical variables using the tertiles of their distribution as cut off values. *Lower class*: values ≤1st tertile. *Intermediate class*: values >1st and ≤2nd tertile. *Upper class*: values >2nd tertile. P: *p* value at Wald test. The Table shows the median values and, in parenthesis, the interquartile range. P: *p* value at Mann-Whitney test.

As an additional result, the Cox analysis of PFS and OS showed that worse prognosis was significantly associated with extension greater than 10 cm in all the models but that including CEA (data not shown); such a result was mainly due to the highly significant association between CEA and disease extension (Table 3).

### 3.3. Biomarkers Modulation during Treatment

**Figure 3:**
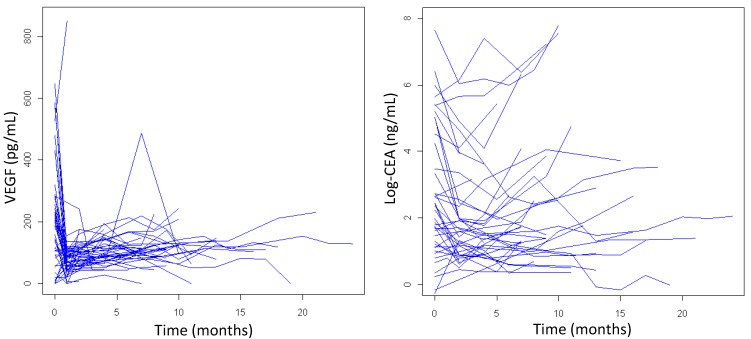
VEGF (left panel) and CEA (log-scale; right panel) modulation during treatment in individual patients.

As shown by plotting individual patients data, VEGF, OPN, SDF-1 tended to sharply decrease until the earlier 3-weeks evaluation before cycle 2, while CEA decreased until the first reassessment after cycle 3; all biomarkers tended to increase thereafter. As an example, Figure 3 plots the individual patients data for VEGF (left panel) and CEA (log-scale; right panel). Such a trend was statistically significant at multivariable mixed effects model analysis (*p* < 0.0001 for VEGF, OPN and CEA; *p =* 0.01 for SDF-1). Time variation was not relevant nor statistically significant as far as PDGF-AB-BB and PDGF-AA were concerned (*p =* 0.476 and 0.542, respectively). There was no significant difference in biomarker modulation during treatment between treatment arms; in contrast, greater disease extension was associated with significantly higher biomarker during treatment levels (data not shown). 

**Figure 4:**
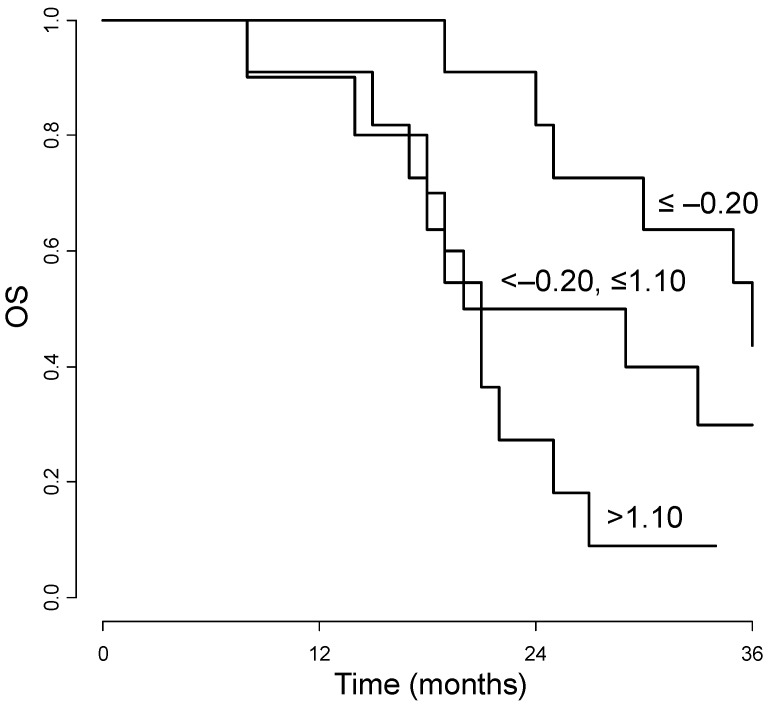
Kaplan-Meier overall survival curves (OS) according to CEA (log-scale) post-nadir variation. The variation was expressed as the difference between the individual levels at last follow-up and at nadir (end of first cycle); the individual differences were categorized according to tertiles (positive difference: CEA increase; negative difference: CEA decrease).

According to the multivariable Cox analyses set up for studying the prognostic association between PFS or OS and biomarker modulation over time, for all the biomarkers the initial decrement (up to the end of first cycle) was not significantly associated with prognosis. As regards the post-nadir modulation CEA increasing levels were found to be significantly related to unfavorable prognosis (0.007 for PFS and *p =* 0.016 for OS). To clarify the reader’s comprehension, Figure 4 shows the OS curves according to CEA post-nadir individual variation (difference between last follow-up and nadir levels). The curves were estimated in the subset of 28 (56%) patients with available data, thus the analysis is less powerful than that performed by the joint use of the mixed effects and Cox models, which were instead estimated on the whole patient data and took into account of the whole individual time profiles. CEA increase (difference > 1.10) was associated with the worst OS (*p =* 0.016). As far as the other biomarkers were concerned, the modulation over time could not be demonstrated as significantly related to the investigated end-points (data not shown).

## 4. Discussion

At present, very little is available in terms of reliable predictive indicators of response and outcome during antiangiogenic treatment, while the most promising biomarkers still need to be independently and prospectively validated in large and randomized trials. VEGF is the most important and studied proangiogenic factor and its action is primarily mediated through VEGF-receptor 2 (VEGFR-2)/Flk-1 [17]. VEGF plasma levels showed prognostic value in some studies, with possible correlation to metastatic potential and disease extension of colorectal cancer [18,19]. In our study, worse prognosis was associated with high baseline VEGF levels, although this correlation did not reach statistical significance. Tissue VEGF expression failed to demonstrate a significant predictive value in the exploratory analysis of 278 out of 813 patients enrolled in the pivotal phase III study of mIFL plus BEV or placebo [20]. More recently, the phase II study of 40 mCRC patients treated with irinotecan, folinic acid and 5-fluorouracil (FOLFIRI regimen) plus BEV showed that elevated Interleukin-8 (IL-8), but not VEGF and VEGFR-2, was associated with disease outcome (PFS) [21]. However, it must be pointed out that VEGF values are dynamic and their changes during treatment might be more important than baseline measurements, particularly as surrogate biomarker in terms of response prediction and progression anticipation [22]. In the initial phase I study of BEV in patients with advanced cancers, Gordon *et al*. [23] reported a decrease of free VEGF and a parallel increase of total concentration, possibly reflecting changes in both free and BEV-bound VEGF or the extensive loading of the extracellular matrix with VEGF in patients with advanced cancers [24]. This methodological observation could explain the significant increase of both VEGF and PIGF serum levels reported previously [24,25]. On the other hand, the use of immunodepleted plasma samples may allow the demonstration of significant decrease of free-VEGF during BEV-based treatment [26]. Thus, the degree of plasma VEGF changes was hypothesised to predict treatment benefits [27].

We showed a significant positive association between baseline VEGF and SDF-1, a small cytokine belonging to the chemokine family named chemokine ligand 12 (CXCL12) and playing an important role in angiogenesis by recruiting endothelial progenitor cells from the bone marrow and stimulating metastases development [28]. This is the first study to demonstrate a significant association of baseline SDF-1 levels with colorectal cancer disease extension, with a trend towards correlation between higher values and worse prognosis during BEV-treatment. In a previous phase II study of sunitinib in hepatocellular carcinoma it was found that elevated plasma levels of SDF-1 were associated with poorer outcome; [29] moreover treatment with BEV of hepatocellular carcinoma determines a decrease of both VEGF and SDF-1 levels [30]. No significant changes of PDGF isoforms were observed and their further exploration as biomarkers in mCRC patients during BEV treatment is not intriguing.

In keeping with the literature, higher baseline CEA levels were associated with poorer prognosis [31,32]. According to ASCO guidelines, CEA is the biomarker of choice for monitoring metastatic CRC treatment, with measurements should be taken at the start of treatment and every 1 to 3 months during treatment [33]. As proposed earlier [34], we showed that CEA kinetics may be used to monitor treatment and potentially predict benefit from BEV-based chemotherapy. Although a CEA flare without PD may occur during the first 4 to 6 weeks of oxaliplatin- or BEV-based chemotherapy [35,36,37]. We performed assays concomitantly with disease reassessments every three cycles.

Although identification of patients who were likely to benefit from antiangiogenic therapy was the main objective of our study, we tried to identify resistance biomarkers associated with disease progression. In this regard, factors originating from the stromal compartment of CRC tissue or proangiogenic/inflammatory chemokines may be the best candidates. In fact, an increase of compensatory angiogenic factors may stimulate new vessels growth in a VEGF-independent manner, sustaining clinical progression through the so called “angiogenic switch”. Kopetz *et al*. recently reported an elevation of PIGF, bFGF, hepatocyte growth factor (HGF) and SDF-1 before evidence of radiological progression in a subset of mCRC patients receiving BEV [21]. In our study, CEA monitoring was the only circulating biomarker with accuracy for identifying patients with worst outcome during continual exposure to BEV. Interestingly, this is the first study to define the priority role of CEA for possible modification of treatment strategy in mCRC patients treated with BEV-containing chemotherapy. Thus, CEA may be the less expensive and most reliable and accurate biomarker to anticipate disease progression in our study and in previous ones [38,39]. 

The main limitations of the present study are constituted by the small sample size and the retrospective, monoinstitutional nature. However, our results were obtained from a prospectively enrolled patients population treated homogeneously into a randomized, open label, phase II trial and all circulating biomarkers, their serial assessment and clinical correlations were pre-specified and scheduled by an ethically-approved ancillary protocol. Despite some limitations, the current study gives insight into the potential role of circulating biomarkers for prognosis assessment and disease monitoring of mCRC patients during first-line BEV-containing chemotherapy. Further prospective studies and potentially cost-saving researches are warranted in this setting, in order to validate additional predictive biomarkers in the knowledge of outcome during angiogenesis inhibition *in vivo*.

## 5. Conclusions

Monitoring angiogenesis biomarkers is a feasible and non-invasive opportunity to study the cancer process. The aim of this study was to investigate the prognostic role of circulating VEGF, PDGF, SDF-1, osteopontin and CEA in mCRC patients treated with BEV-based therapies. This study provides insight into the prognostic meaning of some biomarkers analyzed in terms of PFS and OS in a statistical model that consider the disease extension as an adjustment factor.

We found that CEA and SDF-1 baseline levels were significantly associated with disease extension. In addition, high baseline CEA, SDF-1 and VEGF levels were associated with worse prognosis, but this correlation did not reach statistical significance.

In our series CEA was the only circulating biomarker with accuracy for identifying patients with worst outcome during BEV treatment was CEA. 
